# Supplementary material for: Human leishmaniasis vaccines: Use cases, target population and potential global demand
Source: PLoS Negl Trop Dis. 2021 Sep 21;15(9):e0009742. doi: 10.1371/journal.pntd.0009742 (PMC8486101; doi:10.1371/journal.pntd.0009742)
Supplement: S1 Text — (DOCX) [file pntd.0009742.s001.docx]

**Supplementary Text to the Article “Human Leishmaniasis Vaccines: use cases, target population and potential global demand”**

**DEFINTION OF EPIDEMIOLOGICAL ESTIMATES**

1. **Estimate of population at high risk of VL – used in forecasting demand for prophylactic VL vaccine**

Step 1: identification of alternative sources for global population at risk:

- **WHO’s leishmaniasis country profiles** [1] **integrated with estimates from Pigott et al.** [2] – the estimate includes 14 countries identified by WHO as high burden countries and 11 others with incidence above 1/100k according to the Global Burden of Disease 2019 [3]. Additional validations were performed for the total population of the Indian subcontinent (India, Bangladesh and Nepal) [4] . This selection aims at reflecting countries where VL could be considered a sufficiently high public health burden to justify countries’ investment into the adoption of a vaccine.

|  |  |  |  |  |  |  | Estimated population at risk of VL | | 2019 VL incidence per 100,000, all ages | | |
| --- | --- | --- | --- | --- | --- | --- | --- | --- | --- | --- | --- |
| Country | WHO HIGH RISK | REGION | WB Income Level (2020) | GAVI SUPPORT | DISEASE | VL TRANSMISSION | WHO Estimates | Pigott 2014 | val | upper | lower |
| **South Sudan** | **X** | AFRICA | LIC | X | VL | ANTHROP. | **2'034'944** | 3'749'817 | 46.57 | 68.28 | 30.82 |
| **Sudan** | **X** | AFRICA | LIC | X | CL & VL | ANTHROP. | **8'696'636** | 16'259'580 | 9.63 | 15.05 | 5.59 |
| **Somalia** | **X** | AFRICA | LIC | X | VL | ANTHROP. | **2'377'787** | 2'363'005 | 6.88 | 10.13 | 4.55 |
| **Ethiopia** | **X** | AFRICA | LIC | X | VL |  | **3'168'835** | 36'732'612 | 1.95 | 2.89 | 1.28 |
| **Kenya** | **X** | AFRICA | LMIC | X | VL |  | **3'268'626** | 14'151'164 | 2.16 | 2.93 | 1.56 |
| **Brazil** | **X** | AMERICA | UMIC |  | CL & VL |  | **80'235'408** | 103'739'410 | 2.30 | 3.31 | 1.57 |
| **Georgia** | **X** | EUROPE | UMIC | X | VL |  | **2'593'595** | 1'799'810 | 2.04 | 3.13 | 1.17 |
| **Nepal** | **X** | ASIA | LMIC | X | VL | ANTHROP. | **28'624'296** | 13'864'455 | 0.92 | 1.41 | 0.57 |
| **India** | **X** | ASIA | LMIC | X | CL & VL | ANTHROP. | **130'000'000** | 495'733'890 | 0.59 | 0.92 | 0.34 |
| **Bangladesh** | **X** | ASIA | LMIC | X | VL | ANTHROP. | **30'000'000** | 72'436'664 | 0.22 | 0.34 | 0.13 |
| **Spain** | **X** | EUROPE | HIC |  | VL |  | **37'154'139** | 31'302'114 | 0.12 | 0.20 | 0.06 |
| **China** | **X** | ASIA | UMIC |  | VL | PARTIALLY | **229'718'479** | 205'894'780 | 0.06 | 0.08 | 0.05 |
| **Paraguay** | **X** | AMERICA | UMIC |  | CL & VL |  | **3'060'725** | 2'764'511 | 0.82 | 1.17 | 0.56 |
| **Uganda** | **X** | AFRICA | LIC | X | VL |  |  | **4'603'301** | 0.22 | 0.36 | 0.12 |
| **Central African Rep.** |  | AFRICA | LIC | X | VL |  |  | **1'069'348** | 7.61 | 26.60 | 1.08 |
| **DR Congo** |  | AFRICA | LIC | X | VL |  |  | **12'956'326** | 3.26 | 11.74 | 0.50 |
| **Malawi** |  | AFRICA | LIC | X | VL |  |  | **4'266'093** | NA | NA | NA |
| **Niger** |  | AFRICA | LIC | X | VL |  |  | **2'398'483** | 2.24 | 7.84 | 0.32 |
| **Tanzania** |  | AFRICA | LMIC | X | VL |  |  | **1'575'703** | NA | NA | NA |
| **Eritrea** |  | AFRICA | LIC | X | VL |  |  | **2'665'204** | 2.36 | 5.29 | 0.83 |
| **Chad** |  | AFRICA | LIC | X | VL |  |  | **2'679'550** | 1.11 | 3.51 | 0.18 |
| **Zambia** |  | AFRICA | LMIC | X | VL |  |  | **4'129'548** | 1.22 | 4.43 | 0.18 |
| **Angola** |  | AFRICA | LMIC | X | VL |  |  | **1'276'322** | 1.55 | 4.99 | 0.26 |
| **Kyrgyzstan** |  | ASIA | LMIC | X | VL |  |  | **3'003'517** | 1.12 | 3.87 | 0.19 |
| **Djibouti** |  | AFRICA | LMIC | X | VL |  |  | **652'505** | 4.31 | 6.96 | 2.55 |

- **WHO Technical Report Series** [5] – the estimate includes global population at risk for all leishmaniases for 2010. The totality of this undifferentiated population at risk has been attributed to VL, consistently with the detailed Pigott estimates where the difference between VL and CL at risk populations is minimal.
- **Drug for Neglected Diseases Initiative (DNDi)** [6] - global estimate for Visceral Leishmaniasis for 2009

Step 2: definition of 2018 estimates across the different sources

In order to define comparable estimates, projections of older data point have been created applying the population growth rate for the period 2010-2015 as reported by UN/DESA [7]. The yearly growth rate of 1.8% is calculated as a weighted growth rate based on number of VL cases for 2019 as per the Global Burden of Diseases 2019 (cit.) in the regions where VL is endemic (Asia, Africa and Mediterranean area, Americas). No significant epidemiological change has been assumed.

|  | Growth rate 2010-2015 | Weight (VL cases 2019) |
| --- | --- | --- |
| Africa & Mediterranean region | 2.58% | 48% |
| Asia | 1.04% | 36% |
| Latin America | 1.18% | 16% |
| **Global** | **1.80%** | **100%** |

Step 3: definition of 2018 estimate ranges

Based on the estimates in step 1 and 2, the range for the global population at risk of VL in 2018 has been defined:

| Scenario | Source | 2018 Value |
| --- | --- | --- |
| Upper limit | WHO country estimates and Pigott 2014 | **647 million** |
| Base Case | WHO TRS 2010 | **404 million** |
| Lower Limit | DnDi 2009 | **235 million** |

Step 4: definition of future projections (period 2019-2040)

Starting from the 2018 data point, estimates of the population at risk for the period 2019-2040 have been created by applying a **1.44%** weighted population yearly growth rate for the period 2020-2040 as estimated by UN/DESA (cit.). The growth rate is calculated as the weighted growth rate based on number of cases in 2019 as per the Global Burden of Diseases 2019 (cit.) in the regions where VL is endemic (Asia, Africa and Mediterranean area, Americas). No significant epidemiological change has been assumed for the future.

|  | Growth rate 2020-2040 | Weight (VL cases 2019) |
| --- | --- | --- |
| Africa & Mediterranean region | 2.33% | 48% |
| Asia | 0.59% | 36% |
| Latin America | 0.67% | 16% |
| **Global** | **1.44%** | **100%** |

Step 5: definition of the impact of the introduction of a VL prophylactic vaccine on the population at risk of VL

It is estimated that the introduction of a VL prophylactic vaccine will uniquely impact the size of the population at risk in areas with anthroponotic-only transmission in Asia (India, Bangladesh, Nepal and 19% of the total at risk population in China [8]^[[1]](#footnote-2)^ ) and in Africa [9] (Sudan, South Sudan and Somalia). In those areas the decline of the population is assumed to happen linearly over 4 years starting from 2035 (5 years after the start of the deployment of the vaccine) when the entire population below 20 years is reached. This is under the assumption that those countries will progressively stop treatments/ immunization once all districts have reached elimination (e.g., having achieved reduction of cases below the 1/10,000 per year incidence threshold). The total population at risk of the countries with anthroponotic-only transmission accounts for 245.5 million or approximately 40% of the total population at risk.

1. **Estimate of global VL incidence - used in forecasting demand for a preventative PKDL vaccine**

Step 1: definition of 2019 regional population ranges for the PKDL endemic regions

Estimates are based on the 2019 ranges from the GBD 2019 (cit.) for the 9 PKDL endemic countries (Africa: South Sudan, Sudan, Somalia, Eritrea, Ethiopia, Djibouti - Asia: Nepal, India, Bangladesh). Additional validation were performed based on estimates of new VL cases sourced from Alvar (personal communication) for India and East Africa for 2018 and from Mondal et al. [10] for India for the period 2005-2008.

| Region | Estimate | Source | 2019 Value |
| --- | --- | --- | --- |
| Asia | High | GBD 2019, Alvar | 13801 |
|  | Medium |  | 8771 |
|  | Low |  | 5104 |
| Africa | High | GBD 2019, Alvar, Mondal, | 18091 |
|  | Medium |  | 11959 |
|  | Low |  | 7351 |
| TOTAL 2019 | **High** |  | **31892** |
|  | **Medium** |  | **20730** |
|  | **Low** |  | **12635** |

Step 2: definition of future projections.

The 2019 estimates have been projected through 2040 based on the prevalent population compounded average growth rate (CAGR) for the period 2010-2019 from GBD 2019 (cit.) differentiated for the High, Medium, Low scenarios.

| Region | Scenario | CAGR 2010-2019 |
| --- | --- | --- |
|  | High | -16.48% |
| Asia | Base | -19.41% |
|  | Low | -22.51% |
|  | High | -11.37% |
| Africa | Base | -9.72% |
|  | Low | -9.43% |

For the high and medium scenarios, a wave-shaped incidence curve was applied for the period 2017-2040 reflecting an epidemiological trend with 5 years of decline followed by 5 years of growth, but less accentuated than the cyclical incidence variations induced by El Niño [11] [12] [13], and using the UN DESA regional average annual growth rates (cit.) for the period 2010-2015 (with a positive sign in the growth and a negative sign in the decline years). 2019 is assumed to be in the middle of the declining wave. For the low scenario, a continued decline is instead assumed.

Step 3: definition of the impact of introduction of a VL prophylactic vaccine on VL incidence

In order to assess the impact of the introduction of a VL prophylactic vaccine, the results of the impact modelling performed for India (fully anthroponotic transmission) by Erasmus Medical School has been used [14]. In such model, under the assumption that asymptomatic population also contributes to transmission, the reduction in number of cases following vaccination with a 50% efficacious vaccine (population is 50% less likely to get infected) and capable of covering 100% of the population is the following:

|  | Year 1 | Year 2 | Year 3 |
| --- | --- | --- | --- |
| Reduction of cases | -30% | -50% | -62% |
| As % of vaccine efficacy | -60% | -100% | -124% |

On those bases, and taking into account the proportion of total global population reached (calculated based on the proxy of the introduction sequence of Pneumococcal Conjugate Vaccine in Gavi countries [15] ), the following curve has been applied to estimate the reduction in the number of global VL cases in each of the 3 scenarios:

| Year 1 | Year 2 | Year 3 | Year 4 | Year 5 | Year 6 | Year 7 | Year 8 | Year 9 |
| --- | --- | --- | --- | --- | --- | --- | --- | --- |
| 2.8% | 16.5% | 31.9% | 49.6% | 65.6% | 77.8% | 85.5% | 91.9% | 95.0% |

The reduction in number of cases is applied with one-year delay compared to the year of introduction of the vaccine.

1. **Estimate of global PKDL incidence and prevalence – used in forecasting demand for PKDL therapeutic vaccine**

Step 1: definition of the appropriate VL sequelae rate

Appropriate sequelae rates were estimated for the two regions

| Region | Source | High Sequelae rate | Low Sequelae rate | Average |
| --- | --- | --- | --- | --- |
| Africa | P.Kaye 2019^[[2]](#footnote-3)^ | 25% | 20% | 22.5% |
| Asia | Zijstra 2016 [16] | 20% | 10% | 15% |

Step 2: definition of the PKDL incidence range

Based on the estimates of VL incidence and on the sequelae rates, ranges of PKDL incidence for 2019 and following years were also estimated (the 2019 values are presented in table 10 below:

| Region | Estimate | 2019 Value |
| --- | --- | --- |
| Africa | High | 4070 |
|  | Medium | 2691 |
|  | Low | 1694 |
| Asia | High | 2070 |
|  | Medium | 1316 |
|  | Low | 766 |
| TOTAL 2019 | **High** | **6141** |
|  | **Medium** | **4006** |
|  | **Low** | **2460** |

Step 3: definition of the impact of introduction of a preventative PKDL vaccine on PKDL incidence

Based on an assumption of years roll out of the vaccine to spread over 2 years and on the reduction of cases illustrated for VL, the following curve has been used to estimate the reduction in the number of cases in each of the 3 scenarios:

| Year | Year 1 | Year 2 |
| --- | --- | --- |
| Reduction | 50% | 90% |

The reduction in number of cases is applied with one-year delay compared to the year of introduction of the vaccine (2027).

Step 4: Estimate of global PKDL prevalence

Starting from the estimate of PKDL incidence range as indicated above, an average duration of PKDL lesions of 3.5 years (based on Mondal 2018 [10]) is applied to calculate the total number of active cases in each single year (e.g., the 2021 value will be equal to the sum of the number of new cases in 2018 (half year), 2019, 2020 and 2021). The 2021 values are presented in Table 12 below:

| Region | Estimate | 2021 Value |
| --- | --- | --- |
| Africa | High | 12911 |
|  | Medium | 8658 |
|  | Low | 5466 |
| Asia | High | 6278 |
|  | Medium | 3888 |
|  | Low | 2201 |
| TOTAL 2019 | **High** | **19189** |
|  | **Medium** | **12547** |
|  | **Low** | **7668** |

1. **Estimate of population at high risk of CL – used for the forecasting of demand for a CL prophylactic vaccine**

Step 1: comparison of different sources:

- **WHO country profiles** (cit.) **integrated with Pigott et al.** (cit.) – The estimates include 11 countries identified by WHO having high burden and 22 others with incidence above 5/100k according to the GBD 2019. India (2 states) have also been added taking the total number of countries to 34. This selection aims at reflecting countries where CL could be considered a sufficiently high public health burden to justify countries’ investment into the adoption of a vaccine. Data for 10 countries and the total for the 14 countries in the PAHO region reflect WHO detailed estimates from Leishmaniasis relevant country/regional profiles. Other 9 countries are sourced from Pigott estimates, while for India the estimate corresponds to the population of Rajasthan and Kerala where the diseases is endemic.

|  |  |  |  |  |  | Estimated Population at Risk of CL | | 2019 Cutaneous and mucocutaneous leishmaniasis - national incidence per 100,000 , all ages | | |
| --- | --- | --- | --- | --- | --- | --- | --- | --- | --- | --- |
| Country | WHO High Risk | REGION | WB Income Level (2020) | GAVI SUPPORT | DISEASE | WHO Estimates^3^ | Pigott 2014 | val | upper | lower |
| **Syria** | **x** | ASIA | LIC | X^2^ | CL | **18'502'000** | 20'784'102 | 728.63 | 1'409.58 | 258.37 |
| **Afghanistan** | **x** | ASIA | LIC | X | CL | **10'340'735** | 15'616'552 | 413.90 | 801.91 | 145.24 |
| **Tunisia** | **x** | AFRICA | LMIC |  | CL | **6'018'090** | 9'711'311 | 152.72 | 304.52 | 51.41 |
| **Suriname** |  | AMERICA | UMIC |  | CL |  | 520'165 | 149.60 | 183.56 | 116.94 |
| **Costa Rica** |  | AMERICA | UMIC |  | CL |  | 4'243'499 | 126.91 | 259.81 | 43.76 |
| **Nicaragua** |  | AMERICA | LMIC | X | CL |  | 4'003'127 | 119.08 | 212.65 | 53.75 |
| **Panama** |  | AMERICA | HIC |  | CL |  | 3'308'074 | 103.38 | 353.92 | 4.03 |
| **Libya** |  | AFRICA | UMIC |  | CL |  | **5'716'142** | 98.19 | 218.08 | 25.87 |
| **Iraq** |  | ASIA | UMIC |  | CL |  | **29'738'302** | 84.96 | 184.35 | 26.00 |
| **Yemen** |  | ASIA | LIC | X | CL |  | **20'909'202** | 80.87 | 162.36 | 27.19 |
| **Honduras** |  | AMERICA | LMIC | X | CL |  | 5'488'859 | 77.81 | 116.28 | 43.24 |
| **Algeria** | **x** | AFRICA | LMIC |  | CL | **10'005'224** | 30'969'660 | 66.29 | 130.30 | 23.41 |
| **Peru** | **x** | AMERICA | UMIC |  | CL |  | 20'095'782 | 65.67 | 106.54 | 31.80 |
| **Bolivia** |  | AMERICA | LMIC | X | CL |  | 5'727'962 | 61.50 | 86.39 | 41.19 |
| **Colombia** | **x** | AMERICA | UMIC |  | CL |  | 44'869'432 | 55.09 | 197.48 | 1.50 |
| **Brazil** | **x** | AMERICA | UMIC |  | CL & VL |  | 148'786'750 | 36.04 | 51.57 | 23.46 |
| **Morocco** | **x** | AFRICA | LMIC |  | CL | **6'130'393** | 30'282'374 | 27.15 | 56.05 | 9.58 |
| **Ecuador** |  | AMERICA | UMIC |  | CL |  | 12'469'102 | 26.75 | 54.11 | 8.99 |
| **West Bank^1^** |  | ASIA | LMIC |  | CL |  | **2'842'185** | 26.75 | 54.11 | 8.99 |
| **Gaza Strip^1^** |  | ASIA | LMIC |  | CL |  | **1'515'670** | 26.40 | 60.81 | 6.71 |
| **Saudi Arabia** | **x** | ASIA | HIC |  | CL | **3'590'391** | 21'682'114 | 24.49 | 45.28 | 10.35 |
| **Venezuela** |  | AMERICA | UMIC |  | CL |  | 26'249'248 | 23.96 | 38.01 | 12.50 |
| **Burkina Faso** |  | AFRICA | LIC | X | CL |  | **5'379'299** | 23.21 | 42.76 | 9.40 |
| **Guyana** |  | AMERICA | UMIC | X | CL |  | 651'271 | 16.93 | 76.68 | 0.04 |
| **Turkmenistan** |  | ASIA | UMIC |  | CL |  | **3'957'351** | 14.82 | 38.10 | 2.19 |
| **Sri Lanka** |  | ASIA | LMIC | X | CL |  | **7'250'986** | 12.25 | 36.61 | 0.95 |
| **Guatemala** |  | AMERICA | UMIC |  | CL |  | 8'991'769 | 10.23 | 41.95 | 0.13 |
| **Jordan** |  | ASIA | UMIC |  | CL |  | **6'358'596** | 8.08 | 16.96 | 2.36 |
| **Turkey** | **x** | ASIA | UMIC |  | CL | **41'658'616** | 20'876'026 | 7.46 | 16.74 | 2.18 |
| **Sudan** |  | AFRICA | LIC | X | CL & VL | **37'419'625** | 21'327'576 | 7.64 | 16.78 | 2.06 |
| **Uzbekistan** |  | ASIA | LMIC | X | CL | **15'491'744** | 12'793'034 | 7.66 | 17.08 | 1.63 |
| **Paraguay** |  | AMERICA | UMIC |  | CL & VL |  | 5'209'033 | 7.56 | 15.26 | 2.52 |
| **Pakistan** |  | ASIA | LMIC | X | CL | **86'430'000** | 156'427'700 | 6.80 | 10.21 | 3.99 |
| **Iran** | **x** | ASIA | UMIC |  | CL |  | **60'143'656** | 3.64 | 7.21 | 1.47 |
| **India (2 states)** |  | ASIA | LMIC | X | CL & VL |  | 319'099'420 | 0.03 | 0.04 | 0.02 |
| **PAHO TOTAL** |  |  |  |  |  | **240'000’000** |  |  |  |  |

- **DNDi Disease Profile from 2018** [17] for all cases of leishmaniasis where it is assumed that 100% of the cases will be relevant for CL
- **WHO Technical Report Series 2010** (cit.) the estimate includes global population at risk for all leishmaniases for 2010. Similarly to VL, the totality of this undifferentiated population at risk has been attributed to CL, consistently with the detailed Pigott estimates where the difference between VL and CL at risk populations is minimal.

Step 2: definition of 2018 estimates across the different sources

With the same approach used for VL, projections of older data point have been created applying the population growth rate for the period 2010-2015 as reported by UN/DESA (cit.). The growth rate is a weighted growth rate based on number of 2019 CL cases as per the Global Burden of Diseases 2019 (cit.) in the regions where CL is endemic (Asia, Africa and Mediterranean area, Americas). **The value used is 1.67% yearly growth.** No significant epidemiological change has been assumed.

|  | Growth rate 2010-2015 | Weight (VL cases 2019) |
| --- | --- | --- |
| Africa & Mediterranean region | 2.58% | 38% |
| Asia | 1.04% | 33% |
| Latin America | 1.18% | 29% |
| **Global** | **1.67%** | **100%** |

Step 3: definition of 2018 estimate ranges

Based on the estimates in step 1 and 2, the range for the global population at risk of CL in 2018 has been defined:

| Scenario | Source | 2018 Value |
| --- | --- | --- |
| Upper limit | DnDI 2018 | **1 billion** |
| Base Case | WHO country estimates and Pigott 2014 | **773 million** |
| Lower Limit | WHO TRS 2010 | **399 million** |

Step 4: definition of future projections

Starting from the 2018 data point, estimates of the population at risk for the period 2019-2040 have been created by applying a **1.27%** weighted population yearly growth rate for the period 2020-2040 as estimated by UN/DESA (cit.). The growth rate is a weighted growth rate based on number of CL cases in 2019 as per the Global Burden of Diseases 2019 (cit.) in the regions where CL is endemic (Asia, Africa and Mediterranean area, Americas). No significant epidemiological change has been assumed for the future.

|  | Growth rate 2020-2040 | Weight (VL cases 2019) |
| --- | --- | --- |
| Africa & Mediterranean region | 2.33% | 38% |
| Asia | 0.59% | 33% |
| Latin America | 0.67% | 29% |
| **Global** | **1.27%** | **100%** |

Step 5: definition of the impact of introduction of a vaccine on the population at risk of CL

It is estimated that the vaccine will not have any impact in the reduction of the population at risk of CL the disease being a zoonosis (with the exception of L. tropica)

**DEFINTION OF VACCINE AND IMPLEMENTATION PARAMETERS**

1. **Definition of Vaccine parameters**

Age of administration of the first dose of the vaccine is defined based on the TPP (with no specific age for PKDL) determining the time of the first and eventually subsequent administration.

Efficacy of the vaccine (in term of reduction of number of cases) is defined based on the TPP

Duration of protection of the vaccine is defined based on the TPP informing the number of series required for the vaccine

Number of doses per series is defined based on the TPP

Number of required series is calculated based on the duration of protection and limited to a maximum of 3 series. No vaccine is currently administered with more than 4 series (DTP 3 doses in the first year of life and 3 boosters between 4 and 15 years of age).

| Indication | Age for first dose | Efficacy | Duration of Protection | Nr. Doses per Series | Series |
| --- | --- | --- | --- | --- | --- |
| VL prophylactic | 1 year | 70-95% | 5 years | 2 | 3 (at year 1, 6 and 11) |
| CL prophylactic | 1 year | 70-90% | 5 years | 2 | 3 (at year 1, 6 and 11) |
| VL/CL pro catch-up |  | 70-90% |  | 2 | 1 |
| PKDL therapeutic | NA | 30-90% | Lifelong | 1 | 1 |
| PKDL preventive | NA | 30-90% | Lifelong | 1 | 1 |

Year of licensure and of prequalification (differentiated for VL, CL and PKDL). Under the assumption that the vaccine will be manufactured and commercialized by an experienced manufacturer familiar with the WHO’s prequalification (PQ) processes, a delay of 12 months has been assumed for the time of achievement of the WHO PQ from the time of the first registration.

| Indication | Year of first registration | Year of WHO prequalification |
| --- | --- | --- |
| VL prophylactic | 2029 | 2030 |
| CL prophylactic | 2029 | 2030 |
| PKDL therapeutic | 2027 | NA |
| PKDL preventive | 2027 | NA |

1. **Definition of Implementation parameters**

Coverage proxies are defined for the different modes of delivery:

- Coverage proxy for routine delivery in the first year of life is defined based on WHO & UNICEF Coverage Estimates WUENIC [18] estimates of first dose of measles containing vaccines (MCV1) for the African region
- Coverage proxy for routine delivery via school administration (5 to 14 years of life) is based on HPV coverage estimates (as per Bruni 2016 [19])
- Coverage proxy for routine adult and out-of-school delivery is based on anecdotal evidence from WHO
- Coverage proxy for campaign delivery is based on anecdotal evidence from Gavi and WHO on coverage achieved in Supplementary Immunization Activities (SIAs)
- Primary and Secondary school enrolment rates are based on UNICEF estimates [20] for the African region and used to calculate the coverage of the population in the school period according to this formula:

AVERAGE ((School coverage * Primary Enrollment rate + Out-of-school coverage * (1 – Primary Enrollment rate), (School coverage * Secondary Enrollment rate + Out-of-school coverage * (1 – Secondary Enrollment rate))

Definition of Leishmaniasis vaccine target coverage

On those bases, relevant target coverage estimates – e.g., the coverage that will be reached by the vaccine once fully introduced in the national immunization program of a country - are defined depending on the relevant administration age/es.

| Indication | Delivery | Proxy | Coverage |
| --- | --- | --- | --- |
| VL & CL Prophylactic | Routine at 9 months | MCV1 | 73% |
|  | Routine at 6 years – 11 years | HPV and Primary and Secondary School enrolment rate | 68% |
|  | Routine for adults | Anecdotal | 45% |
|  | Campaigns delivery (including catch-up) | SIA analyses | 90% |
| PKDL Therapeutic & Preventive | Following delivery of VL or PKDL treatment | 100% in Indian subcontinent  85% in Africa | 93% |

Definition of the standard sequence of country vaccine routine introductions (uptake curve) – looking at the status of the countries most likely to introduce the vaccine, 21 of the 25 countries (84%) which are likely targets for the introduction of a VL vaccine and 13 of the 34 countries (38%) which are likely targets for a CL vaccine are or were eligible. The sequence of introduction of PCV (vaccine rolled-out in the largest majority of countries with no relevant supply of funding constraints) in the Gavi countries is selected as the one providing the most accurate proxy for the sequence (number of country introductions per year) of introductions into routine immunization in the targeted countries. The curve indicates the growing share of the targeted population reachable by the vaccine as result of country decisions to introduce the vaccine in their immunization schedule.

|  | 2009-2010 | 2011 | 2012 | 2013 | 2014 | 2015 | 2016 |
| --- | --- | --- | --- | --- | --- | --- | --- |
| Countries in the year | 3 | 13 | 8 | 14 | 8 | 8 | 3 |
| Total cumulative | 3 | 16 | 24 | 38 | 46 | 54 | 57 |
| % of total (nr. 62)^1^ | 4.8% | 25.8% | 38.7% | 61.3% | 74.2% | 87.1% | 91.9% |

Wastage rate: 25% based on WHO standard guidance for a low-multidose (5 or 10 doses) presentation

Buffer stocks: as per Gavi operational forecast: every year 25% of change in total demand

**DOSES CALCULATION**

1. **Definition of target population for different indications and delivery strategy**

Definition of the target population for catch-up campaigns for the prophylaxis against VL and CL

It is assumed that each country will perform a catch-up campaign at start of the program to reach a large portion of the population at risk that will not be otherwise reached with the start of routine immunization (because of older age). On the base of the age distribution of the cases (as per GBD 2019 - cit.) the following target populations are assumed for catch-up:

| Indication | Age distribution | Target age for Catch-Up |
| --- | --- | --- |
| Prophylaxis of VL | Based on VL cases:  30% - 0 to 4 years  35% - 5 to 14 years  35% - 15 years and above | **5 to 14 years**  Catch-up in school to be followed by regular start in EPI at 1 year of age |
| Prophylaxis of CL | Based on CL cases  2% - 0 to 4 years  15% - 5 to 14 years  59% - 15 to 49 years  24% - 50 years and above | **5 to 29 years**  SIA to be followed by start in EPI at 1 year of age |

Definition of the target population for routine immunization

For each year from 2018 through 2040, the population targeted by the vaccine under the various indications is defined as follow:

- **VL**: the total population at risk of VL has been split in age groups based on age distribution as per UN/DESA (cit.) to define the size of the age groups targeted based on the selected routine vaccination strategy and the appropriate coverage level/s (see above the implementation parameter section). Based on the schedules (number of series) indicated in the TPP, it is assumed that one cohort (at 1 year of age) will be targeted in the 0 to 4 years of age group and two cohorts (at 6 at 11 years of age) will be targeted in the 5 to 14 years of age group. Cohort populations are assumed to be equally distributed within the age range.

|  | Population distribution (UN/DESA) |
| --- | --- |
| PSAC (0-4 years) | 9% |
| SAC (5-14 years) | 18% |
| Adolescents & Young adults (15-29 years) | 25% |
| Adults and Elderly (above 29 years) | 48% |

- **CL**: the total population at risk of CL has been split in age groups based on age distribution as per UN/DESA (cit.) to deine the size of the age groups targeted based on the selected routine vaccination strategy and the appropriate coverage level/s (see above the implementation parameter section). **Based on the targeted countries population, at risk a reduction factor of 28.7% is applied to reflect the populations that will be already reached for VL vaccination in India, Sudan, Brazil & Paraguay (see estimates of VL population at risk section)**. Based on the schedule (number of series) indicated in the TPP it is assumed that one cohort (at 1 year of age) will be targeted in the 0 to 4 years of age group and two cohorts (at 6 at 11 years of age) will be targeted in the 5 to 14 years of age group. Cohort populations are assumed to be equally distributed within the age range.

Definition of the target population in the treatment and prevention of PKDL:

- **PKDL Therapeutic**: under the assumption that the total PKDL affected population will be targeted, the calculated global number (see above in the estimate of PKDL incidence and prevalence section) is considered
- **PKDL Preventive**: under the assumption that the total VL cases will be targeted, the calculated global number (see above in the estimate of VL incidence section) is considered

1. **Calculation of doses required**

Before taking into account the time of introduction of the vaccine in the different indication or the programmatic requirements, for each year from 2018 through 2040, the following calculations are performed

- Calculation of **“coverable” population**:
  - VL and CL catch up: for the age groups targeted (as defined above) the SIA coverage is applied to define the maximum number of people that could be reached as per the formula:

“target population x SIA coverage”

- - VL and CL routine: for each cohort the appropriate coverage (as defined above) is applied to the target population to define the number of people that could be reached as per the formula:

“target population x age specific coverage”

- - For PKDL: a delivery post VL/PKDL treatment is foreseen, hence no age specific coverage will be applied but the leishmaniasis treatment coverage (DNDi annectoctal) as per the formula:

“target population x leishmaniasis treatment coverage”

- Calculation of **100% Administrable doses:**
  - Based on the number of series required in each delivery strategy/indication, on doses per series (as defined above) and on the coverable population (as defined above) the number of doses required to vaccinate the population that can be reached by the immunization system is calculated as per the formula:

“coverable population x number of series x doses per series”

1. **Calculation of potential demand**

For each indication, the potential number of doses required is calculated as follow

- Definition of the country uptake:
  - For the catch-up (VL & CL only) the activity is one-off, therefore the country uptake curve (as defined above in the implementation parameters section) is used, via the calculation of the yearly increase (vs. the cumulative number), to define the portion of the total population caught in a specific year. The start of the catch-up campaigns is defined based on the introduction date assumption (defined above in vaccine parameters section).
  - For the routine program (VL & CL only) the country uptake curve (as defined above in the implementation parameters section) is used for each of the indications to define the proportion of the 100% administrable doses that will be reached. The start of the roll-out in routine is defined based on the introduction date assumption (defined above in vaccine parameters section). The uptake curve is applied unmodified for the PSAC that are reached every year since the introduction of the vaccine and with a delay for the two administrations foreseen for SAC to discount the fact that catch-up campaigns initially cover those populations. The delay accounts for the fact that the first booster will be administered from year 3 and the second booster from year 5 and results in the following modified uptake curve for SAC:

| Year 1 | Year 2 | Year 3 | Year 4 | Year 5 | Year 6 | Year 7 | Year 8 | Year 9 | Year 10 | Year 11 | Year 12 |
| --- | --- | --- | --- | --- | --- | --- | --- | --- | --- | --- | --- |
| 0% | 0% | 2% | 13% | 22% | 44% | 56% | 74% | 83% | 94% | 96% | 100% |

- - For the ongoing prevention and treatment of PKDL, the country uptake curve (as defined above) is applied for each of the indications based on the year of registration (as defined above) defining the proportion of the 100% administrable doses that will be reached.
- Calculation of the doses administered: based on the selected uptake curve the total number of doses administered is calculated for each year according to the following formula:

“uptake x target population”

- Calculation of the wastage: for each year the standard % wastage rate (as defined above) is applied to the administered doses according to the following formula:

“administered doses x (1 + wastage rate)”

- Calculation of the buffer stock: for each year the required buffer stock is calculated according to the formula:

“administered doses year^x^ – administered doses year^(x-1)^ x buffer stock rate”

- Calculation of the total number of doses required: for each year, the total number of doses whose production will be required to fulfill the emerging demand is calculated according to the following formula:

“administered doses + wastage + buffer stock”

**REFERENCES**

1. Leishmaniasis country profiles. [cited 3 Aug 2021]. Available: http://who-dev.essi.upc.edu/who/leishmaniasis.html

2. Pigott DM, Sumiko Mekaru HR, Gething PW, George DB, Myers MF, Reithinger R, et al. Global Distribution Maps of the Leishmaniases. Elife. 2014;2010: 1–21. doi:10.7554/eLife.02851

3. Abbafati C, Machado DB, Cislaghi B, Salman OM, Karanikolos M, McKee M, et al. Global burden of 369 diseases and injuries in 204 countries and territories, 1990–2019: a systematic analysis for the Global Burden of Disease Study 2019. Lancet. 2020;396: 1204–1222. doi:10.1016/S0140-6736(20)30925-9

4. Karunaweera ND, Ferreira MU. Leishmaniasis: Current challenges and prospects for elimination with special focus on the South Asian region. Parasitology. 2018;145: 425–429. doi:10.1017/S0031182018000471

5. World Health Organization. Control of the leishmaniases. World Health Organ Tech Rep Ser. 2010; 22–26.

6. Drug for Neglected Disease Initiative. DNDi - NewsLetter. [cited 2 Jan 2020]. Available: https://www.dndi.org/newsletters/n18/4_1.php

7. United Nations - Department for Economic and Social Affairs. World Population Prospects - Population Division - United Nations. In: World Population Prospects 2019 [Internet]. [cited 2 Jan 2020]. Available: https://population.un.org/wpp/Download/Standard/Population/

8. Lun ZR, Wu MS, Chen YF, Wang JY, Zhou XN, Liao LF, et al. Visceral leishmaniasis in China: An endemic disease under Control. Clin Microbiol Rev. 2015;28: 987–1004. doi:10.1128/CMR.00080-14

9. Postigo JAR. Leishmaniasis in the World Health Organization Eastern Mediterranean Region. Int J Antimicrob Agents. 2010;36: S62–S65. doi:10.1016/J.IJANTIMICAG.2010.06.023

10. Mondal D, Bern C, Ghosh D, Rashid M, Molina R, Chowdhury R, et al. Quantifying the infectiousness of post-kala-azar dermal leishmaniasis toward sand flies. Clin Infect Dis. 2019;69: 251–258. doi:10.1093/cid/ciy891

11. Leta S, Dao THT, Mesele F, Alemayehu G. Visceral Leishmaniasis in Ethiopia: An Evolving Disease. PLoS Negl Trop Dis. 2014. doi:10.1371/journal.pntd.0003131

12. Neto AB da S, de Oliveira EF, Encina CCC, de Figueiredo HR, Filho ACP, de Oliveira AG. Effects of El Niño-Southern oscillation on human visceral leishmaniasis in the Brazilian State of Mato Grosso do Sul. Mem Inst Oswaldo Cruz. 2020;115: 1–8. doi:10.1590/0074-02760190298

13. Deb RM, Stanton MC, Foster GM, Das Gupta RK, Roy N, Das P, et al. Visceral leishmaniasis cyclical trends in Bihar, India – Implications for the elimination programme. Gates Open Res. 2018. doi:10.12688/gatesopenres.12793.1

14. Rutte EA Le, Coffeng LE, Malvolti S, Kaye PM, de Vlas SJ. The potential impact of human visceral leishmaniasis vaccines on population incidence. 2020. doi:10.1101/2020.05.05.20090480

15. Johns Hopkins Bloomberg School of Public Health International Vaccine Access Center (IVAC). A report on current global access to new childhood vaccines. 2019.

16. Zijlstra EE, Alves F, Rijal S, Arana B, Alvar J. Post-kala-azar dermal leishmaniasis in the Indian subcontinent: A threat to the South-East Asia Region Kala-azar Elimination Programme. PLoS Neglected Tropical Diseases. 2017. doi:10.1371/journal.pntd.0005877

17. Drug for Neglected Disease Initiative. About Leishmaniasis – DNDi. 2019 [cited 31 Dec 2019]. Available: https://www.dndi.org/diseases-projects/leishmaniasis/

18. WHO UNICEF coverage estimates WHO World Health Organization: Immunization, Vaccines And Biologicals. Vaccine preventable diseases Vaccines monitoring system 2020 Global Summary Reference Time Series: DTP3. [cited 2 Aug 2021]. Available: https://apps.who.int/immunization_monitoring/globalsummary/timeseries/tswucoveragedtp3.html

19. Bruni L, Diaz M, Barrionuevo-Rosas L, Herrero R, Bray F, Bosch FX, et al. Global estimates of human papillomavirus vaccination coverage by region and income level: a pooled analysis. Lancet Glob Heal. 2016;4: e453–e463. doi:10.1016/S2214-109X(16)30099-7

20. Primary School Age Education - UNICEF DATA. [cited 2 Aug 2021]. Available: https://data.unicef.org/topic/education/primary-education/

**LEGEND**

Table 1 (line 11): Overview of key parameters for the 25 VL endemic countries modeled – in red values used in the estimate of the population at risk of VL

Table 2 (line 23): 2010-2019 Growth rate calculation for VL population at-risk

Table 3 (line 26): Estimates of total population at risk for VL in 25 endemic countries for 2018

Table 4 (line 32): 2020-2040 Growth rate calculation for VL population at-risk

Table 5 (line 47): Estimates of VL incidence for 2019

Table 6 (line 51): VL population compounded average growth rate (CAGR) for the period 2010-2019

Table 7 (line 64): Impact of the introduction of a prophylactic VL vaccine on VL incidence

Table 8 (line 68): Global % reduction of VL cases following the introduction of a VL prophylactic vaccine

Table 9 (line 76): Calculation of average VL sequelae rate

Table 10 (line 80): Estimates of PKDL incidence for 2019

Table 11 (line 84): Impact of the introduction of a preventative PKDL vaccine on PKDL incidence

Table 12 (line 91): Estimates of PKDL prevalence for 2021

Table 13 (line 101): Overview of key parameters for the 34 CL endemic countries modeled – in red values used in the estimate of the population at risk of VL, cell shaded in green indicate countries in the PAHO region - 1: West Bank and Gaza Strip estimates are separated; 2: Syria included into Gavi supported countries; 3: total consolidated estimates for PAHO countries instead of individual country estimates

Table 14 (line 112): 2010-2019 Growth rate calculation for CL population at-risk

Table 15 (line 115): Estimates of total population at risk for CL in 34 endemic countries for 2018

Table 16 (line 135): Main TPP parameters

Table 17 (line 139): Year of first marketing authorization and of WHO prequalification

Table 18 (line 156): Target coverage for the different ages of administration

Table 19 (line 163): Sequence of Pneumococcal Conjugate Vaccine introductions in Gavi supported countries (1: total number of countries limited to 62 based on Gavi’s policy limiting support for new introductions to countries with DTP3 coverage > 75%)

Table 20 (line 173): VL and CL target population estimates for catch-up campaigns

Table 21 (line 181): Population age distribution

Table 22 (line 228): Proportion of SAC reached into routine immunization (discounting the proportion already reached by the catch-up campaign at start)

1. In China, three epidemiological types of VL have been described: anthroponotic VL (AVL), mountain-type zoonotic VL (MT-ZVL), and desert-type ZVL (DT-ZVL). AVL is present endemically only in Xinijang where it coexists with MT-VTL. The weight of AVL on the VL total cases in China has been assumed being half of the VL cases in the Xinjinag region as reported in the article. [↑](#footnote-ref-2)
2. Annectoctal discussion on June 2019 [↑](#footnote-ref-3)
