# Supplementary material for: Human leishmaniasis vaccines: Use cases, target population and potential global demand
Source: PLoS Negl Trop Dis. 2021 Sep 21;15(9):e0009742. doi: 10.1371/journal.pntd.0009742 (PMC8486101; doi:10.1371/journal.pntd.0009742)
Supplement: Attachment — Submitted filename: Malvolti et al_Human leishmaniasis vac demand RespLett.docx [file pntd.0009742.s002.docx]

Dear Editors of PLOS Neglected Diseases,

We are hereby coming back on our submission (PNTD-D-21-00594) “Human leishmaniasis vaccines: use cases, target population and potential global demand” with the requested responses to the comments from the reviewers and a description to the changes in the manuscript (highlighted in yellow in the “marked” version).

**Criteria Required for Acceptance**

Reviewer nr. 1 – while no request of change was indicated, we valued very much the comment and opted for replacing the point estimates for the demand for both CL and VL with ranges that reflect different estimates of the population at risk (ref. line 419 and following for the description of the scenarios simulated)

Reviewer nr. 2 – no comment required

Reviewer nr. 3 – we moved and expanded the description of the forecasting methodology employed (line 156 and following – line 146 and following in the older version), more clearly align to the subsequent sections of the manuscript and more clearly linked to figure 1 that provides the graphical representation of the approach.

**Analysis matching the plan and results clearly presented**

Reviewer nr. 1 – no comment required

Reviewer nr. 2 – no comment required

Reviewer nr. 3 – with reference to the control strategies for Kala Azar in the Indian subcontinent and the impact of vaccine hesitancy we have simulated a more conservative scenario (reg. line 419 and following). While at the moment data does not exist that allows to quantify the impact for those variables, we believe the a “generic” more conservative scenario can provide a directional view of how demand may shape if control were to be more successful or hesitancy impacting more on the demand for the vaccine.

With reference to the overlapping VL and CL prevalence we added detailed explanation of the methodology employed to calculate the 29% value used (line 378 and following).

Finally, with refence to the limitations of the model, those are captured from line 511 onwards. We realized that this was not clearly pointed out and we added a sentence to point the reader to that part of the paper when looking for the main limitation of the analysis.

**Conclusions**

Reviewer nr. 1 – no comment required

Reviewer nr. 2 – no comment required

Reviewer nr. 3 – in order to provide a more nuanced view of the potential demand and avoiding giving an incorrect sense of precision/over-optimism, we have now included a more conservative and more optimistic scenario that refer to the different at-risk population estimates available (section “Estimating the target population” starting from line 179 for description of methods and sources and line 371 and following for the results). Results are now presented as ranges allowing for a more nuanced interpretation by the reader.

**Summary and general comment**

Reviewer nr. 1 – on the comment re. India and other countries subnational presence of the disease, it is confirmed that the at-risk population is normally not corresponding to the entire country population based on available sources estimates (ref. line 190 and following for population at-risk of VL / line 228 and following for population at-risk of CL; for India lacking specific WHO estimates for CL only the population of two states have been used re. line 231). Nonetheless, recognizing that those estimates could still be optimistic and that more targeted block strategies can be employed, we developed a more conservative scenario (as described in line 419 and following where the total at-risk population for VL and CL is reduced respectively by 40% and 48%)

With reference to the justification of the therapeutic vaccine for PKDL instead of VL we have expanded our explanation of the rationale in line 128 and following.

With reference to the impact of COVID-19 vaccine on future vaccine development landscape, we believe that the awareness on the importance of the value of vaccines is going to increase with potential benefits for vaccine development programs. Whether this will translate in additional investment in the long-run for all diseases, and, in particular, for NTDs, is a question, and past experiences (SARS-CoV-1 and H1N1) do not necessarily speak for a positive long-term result. The unprecedented nature of the COVID-19 pandemic may change this; however, we feel too premature to include a positive statement on the matter in this manuscript.

With reference to the source for number of cases and DALYs we have clarified that reference number 2 was the source for both those data (line 94 and 95)

With reference to the evidence of VL transmission via L.donovani can be zoonotic, in view of the conflicting evidence we have removed the reference to L.donovani (line 98)

With reference to the immunity against re-infection for CL (line 119)

With reference to the evolution into MCL we slightly modified the sentence starting on line 120 to more precisely portrait the pathogenesis.

Reviewer nr. 2 – with reference to the possible underestimate of the CL at-risk population because current estimates not reflective of migration flows, and to the fact that the catchment areas may expand to whole states for programmatic ease / targeting of asymptomatic population, we have also developed a higher scenario (line 371 and following for the methods and line 419 and following for results) that is meant to represent a broader geographical reach of the disease.

With reference to the different impact of the vaccine in VL endemic areas where transmission is zoonotic, we have clarified the approach that is in fact in line with the comment (line 287 and following)

With reference to the use cases and potential therapeutic use of VL and CL vaccine for other co-infections, those use cases were deemed by the experts consulted as marginally important for this high-level analysis of demand.

With reference to the different vaccination schedules (prime-booster regimes vs. single dose) a specific scenario is included in the manuscript to account for this uncertainty (line 405 and following)

With reference to the role those different incentives may play for manufacturers specific reference to priority review vouchers and advanced market commitments have been added (line 489 and 490)

Finally, with reference to the rationale for a specific PKDL vaccine additional information are provided (line 493)

Reviewer nr. 3 – no comment required
